# Nonlinear Optical Investigation of Microbial Chromoproteins

**DOI:** 10.3389/fpls.2020.547818

**Published:** 2020-10-21

**Authors:** Szilvia Krekic, Tomás Zakar, Zoltán Gombos, Sándor Valkai, Mark Mero, László Zimányi, Zsuzsanna Heiner, András Dér

**Affiliations:** ^1^Institute of Biophysics, Biological Research Centre, Szeged, Hungary; ^2^Doctoral School of Multidisciplinary Medical Sciences, University of Szeged, Szeged, Hungary; ^3^Institute of Plant Biology, Biological Research Centre, Szeged, Hungary; ^4^Max Born Institute for Nonlinear Optics and Short Pulse Spectroscopy, Berlin, Germany; ^5^School of Analytical Sciences Adlershof, Humboldt-Universität zu Berlin, Berlin, Germany

**Keywords:** Z-scan, bacteriorhodopsin, photoactive yellow protein, nonlinear refractive index, saturable absorption, photo-induced refractive index change

## Abstract

Membrane-bound or cytosolic light-sensitive proteins, playing a crucial role in energy- and signal-transduction processes of various photosynthetic microorganisms, have been optimized for sensing or harvesting light by myriads of years of evolution. Upon absorption of a photon, they undergo a usually cyclic reaction series of conformations, and the accompanying spectro-kinetic events assign robust nonlinear optical (NLO) properties for these chromoproteins. During recent years, they have attracted a considerable interest among researchers of the applied optics community as well, where finding the appropriate NLO material for a particular application is a pivotal task. Potential applications have emerged in various branches of photonics, including optical information storage and processing, higher-harmonic and white-light continuum generation, or biosensorics. In our earlier work, we also raised the possibility of using chromoproteins, such as bacteriorhodopsin (bR), as building blocks for the active elements of integrated optical (IO) circuits, where several organic and inorganic photonic materials have been considered as active components, but so far none of them has been deemed ideal for the purpose. In the current study, we investigate the linear and NLO properties of biofilms made of photoactive yellow protein (PYP) and bR. The kinetics of the photoreactions are monitored by time-resolved absorption experiments, while the refractive index of the films and its light-induced changes are measured using the Optical Waveguide Lightmode Spectroscopy (OWLS) and Z-scan techniques, respectively. The nonlinear refractive index and the refractive index change of both protein films were determined in the green spectral range in a wide range of intensities and at various laser repetition rates. The nonlinear refractive index and refractive index change of PYP were compared to those of bR, with respect to photonics applications. Our results imply that the NLO properties of these proteins make them promising candidates for utilization in applied photonics, and they should be considered as valid alternatives for active components of IO circuits.

## Introduction

Ubiquitous applications of photonics and optoelectronics are now penetrating into diverse areas from everyday life to the most advanced scientific disciplines, such as optical communication, data processing and storage, quantum computing, holography, just to mention a few. Key elements of photonic devices are the so-called nonlinear optical (NLO) materials, which can actively modify light propagation or store optical information. Examples for the use of NLO materials in photonics ranges from all-optical signal processing (Willner et al., 2014), to all-optical switching (Chai et al., 2017), optical filtering (Dini et al., 2016), with the list of applications continuously growing. To this end, one of the biggest challenges is finding materials with optimal NLO characteristics that could be applied in photonic devices. The development and characterization of NLO materials (such as nonlinear crystals or chalcogenide glasses) (Adair et al., 1989; Eggleton et al., 2011) is in current progress, because the diverse applications require special solutions. E.g., several aspects must be taken into account when selecting a material for an IO application, such as mechanical stability, re-excitability, sensitivity, but most importantly, the material has to have a large refractive index change induced by an outer stimulus of an electric field or light. Amongst others, the application of molecules possessing π-conjugated electron systems have been most favored, since they show a high (third-order) optical polarizability, usually without two-photon losses (Hales et al., 2010; Haque and Nelson, 2010; Hu et al., 2017). However, various problems concerning their robustness and incorporation into solid matrices for practical applications are still to be solved. On the other hand, natural π-conjugated materials, such as chromoproteins are readily available (Clays et al., 2001; Fábián et al., 2011). Chromoproteins have been perfected by evolution for billions of years for utilizing light as a source of energy or information. Their protein matrix stabilizes their chromophores, and fine-tunes their optical properties. At the same time, the application of proteins as NLO materials in photonics also raises non-trivial technical problems. Building up stable hybrid structures of the protein and the passive substrate (such as a thin film of chromoprotein on a photonic circuit), and a thorough characterization of their NLO properties is inevitable, as well as their optimization for a particular application, such as IO switching, or other optical information processing tasks.

One of the most investigated candidates in the field is the protein bacteriorhodopsin (bR), while other proteins, such as photoactive yellow protein (PYP) have recently also been considered as IO active materials (Krekic et al., 2019). Their molecular and bulk optical properties (e.g., linear and nonlinear polarizabilities, and their refractive index and its light-induced changes) are of high interest from the point of view of potential optoelectronic applications of chromoproteins, in general.

Bacteriorhodopsin is a membrane protein (embedded in quasi-crystalline lipid-protein patches, the so-called purple membranes) first discovered in the archaeon *Halobacterium salinarum* (Oesterhelt and Stoeckenius, 1971; Lanyi, 2004). It consists of seven-transmembrane alpha helices to which an all-trans retinal is covalently attached through a protonated Schiff base. bR is widely known to be the simplest light-driven proton pump (Oesterhelt and Stoeckenius, 1973), hence considered as a model for more complicated systems. One of the protein’s most important characteristics is its photocycle. Upon light absorption, bR enters a reaction cycle, going through quasi-stable intermediate states (K, L, M, N, O) in a matter of milliseconds in solution, before returning to the initial state. Each of the intermediary states possesses characteristic absorption spectra, distinct from the initial state’s spectrum. The difference in absorption spectra between the intermediate states and the initial state indicates a difference in refractive indices according to the Kramers–Kronig relations (Nussenzveig, 1972; Wooten, 2013).

Photoactive yellow protein is a water-soluble protein present in purple sulfur photosynthetic bacteria (Meyer, 1985), much smaller than bR-containing purple membrane patches (Oesterhelt and Stoeckenius, 1971; Meyer, 1985). This makes PYP a promising candidate for incorporation into IO passive structures (e.g., in porous silicon), where membrane-bound bR cannot be used due to its larger particle size. When excited with blue light, PYP enters its photocycle (Meyer et al., 1987), which standardly consists of four intermediates (pR_1_, pR_2_, pB_1_, pB_2_) and takes place in a matter of milliseconds in solution.

To fully consider a protein in IO applications, a conclusive optical characterization is needed. The utility of NLO materials for optical communication is highly dependent on the magnitude of the refractive index change that can be induced in the material at hand. The larger the associated change in the complex refractive index, the larger the amplitude and phase modulation effect will be. When combining NLO materials with passive components, the size and energy consumption also depend on the NLO response, hence a larger refractive index change is preferred (Miller, 2010; Sasikala and Chitra, 2018).

In dried films of chromoproteins the light-induced refractive index changes are orders of magnitude higher than in suspensions, where, due to the overwhelming excess of water (ca. 55 M water to a few 100 μM of protein in the densest suspensions), the refractive index of water is dominating (Heiner and Osvay, 2009). Hence, as far as photonic applications of bR utilizing light-induced optical changes are concerned, dried films (usually under controlled humidity), are used (Dér and Keszthelyi, 2001; Vsevolodov, 2012 and references therein). Exceptions are only the few applications that are based on absorption instead of refractive index changes (Stuart et al., 2001). From the point of view of technical applications, on the other hand, using dried films is convenient due to their form and stability. Combining dried films of proteins with IO passive structures is a solvable task (see the section “Materials and Methods”), and in such samples proteins maintain their optical properties for a long time (several decades) (Váró and Keszthelyi, 1983; Dér and Keszthelyi, 2001; Vsevolodov, 2012).

One should note, however, that the photocycles of both pigments do depend on the relative humidity of the films (Váró and Keszthelyi, 1983; van der Horst et al., 2005). The general rule of thumb is that at high-enough relative humidity (>80–90%), the photocycles are close to the native ones, while at moderate relative humidity (between ca. 30–50% to 80%), such transitions that accompany large-scale conformational changes (e.g., the ones following M formation in the bR, or those after pB_1_ in PYP) are hindered, hence the rate-limiting steps become slower. The reason for this phenomenon is that protein conformational flexibility is decreasing by lowering relative humidity (Fitter et al., 1999). Further lowering relative humidity values leads to more serious truncation of the photocycles (Váró and Keszthelyi, 1983; van der Horst et al., 2005). Technical applications (considered so far for bR only) normally use the higher end of moderate humidity range, as a reasonable trade-off between maximizing **Δ*n*** and having a decent photocycle kinetics (Dér and Keszthelyi, 2001; Vsevolodov, 2012). Proteins in this range show an overall slower photocycle than native proteins, which might appear to be less favorable for some photonic applications, however, both proteins have fast (sub-picosecond to picosecond) transitions in the beginning of their photocycles, which are unaffected by humidity in bR (Colonna et al., 2005), and probably in PYP, too (see fast kinetics detected in crystallized PYP (Yeremenko et al., 2006; Schotte et al., 2012; Tenboer et al., 2014), but yet to be characterized in PYP dry samples). On the other hand, the first intermediate states of both the bR and PYP photocycles can be driven back by fast, light-induced reactions to the ground state (Balashov, 1995; Joshi et al., 2005; Tóth-Boconádi et al., 2006, 2010), allowing a rapid, light-controlled manipulation of refractive index kinetics of the films. The lifetime of various types of dried bR films is known to be in the range of several decades, appropriate for technical applications (Dér and Keszthelyi, 2001).

In the past decades, several research articles have demonstrated the dried bR film’s large light-induced refractive index change (****Δ***n***), and its utilization in optical switching experiments and light modulation (Ormos et al., 2002; Fábián et al., 2011; Mathesz et al., 2013). bR also shows a large hyperpolarizability, important in frequency-doubling experiments, investigated by NLO techniques (Naskali et al., 2014). The nonlinear refractive index value ***n***_**2**_ of bR-containing samples has been extensively investigated previously under various experimental conditions, in most of the cases using the so-called Z-scan technique (Song et al., 1993; Aranda et al., 1995; Jeganathan et al., 2017). The spectro-kinetic properties and light-induced refractive index changes have also been discussed (van der Horst et al., 2005; Krekic et al., 2019), which further encourages investigation of the protein’s NLO properties. There have been a few publications dealing with the nonlinear refractive index of different PYP samples, e.g., embedded in thick polyacrylamide matrices (Vanhanen et al., 1998; Leppanen et al., 1999) or adsorbed to poly-methyl-methacrylate microspheres (Lee et al., 2018), however, the results are hard to reconcile due to the different samples and the various methods applied (Z-scan, Michelson-interferometer, and hyperspectral quantitative phase imaging). No experiments were done so far on a thin-film PYP sample and with a pulsed Z-scan setup.

The single-beam Z-scan technique is a popular method for the characterization of the optical nonlinearities of a wide variety of materials (Sheik-Bahae et al., 1990), including organic and even protein samples. During a Z-scan, the sample is moved through the laser focus and the power or energy transmitted through an aperture placed behind the focus is measured as a function of the sample position, ***z***. When the aperture is small, a closed-aperture trace is obtained, yielding the magnitude and sign of ***n***_**2**_. When the aperture is removed or open, an open-aperture trace is measured, providing the magnitude of nonlinear absorption coefficients. While the experimental realization of the technique is simple, determining all the NLO mechanisms causing a particular Z-scan trace is often not possible without performing additional measurements. For example, the photocycle of bR and the related complex kinetics make the interpretation of the associated Z-scan data difficult (Kir’Yanov et al., 2000).

Here, we report on the NLO properties of a new type of thin bR and PYP films, in the light of possible optoelectronic applications of these proteins. The films were experimentally characterized through OWLS, absorption kinetics and Z-scan measurements. The linear and nonlinear refractive index, and the formation and decay times of the respective intermediate states in the photocycle of the two proteins were determined. The nonlinear refractive index was measured using the Z-scan technique with both 543-nm or 405-nm continuous wave (CW) and 514-nm pulsed laser illumination at varying repetition rates and a wide range of intensities. The determined **Δ*n*** and ***n***_**2**_ values of bR are compared to previously published data, as well as to the results obtained for PYP, where prior data on NLO properties are scarce. The results are considered to have important implications from the perspective of photonic applications – the future utilization of these biomaterials in film format for integrated optical switching and signal processing experiments. Compared to non-organic NLO materials currently used and researched for such purposes, biological materials, such as bR and PYP, offer a readily available, cost-effective alternative, which is also more versatile depending on the form and environment of the protein building blocks.

## Materials and Methods

### Sample Preparation

Bacteriorhodopsin was prepared according to the standard procedure: purple membranes separated from strain R1M1 of *Halobacterium salinarum* were prepared as described in Oesterhelt and Stoeckenius (1971). Purple membranes contain 25% lipids and 75% bR, as the sole protein constituent in the preparation (Oesterhelt and Stoeckenius, 1974). (For the sake of simplicity, we refer to this membrane-bound form of the protein as bR, throughout the text.) Wild-type PYP apoprotein was overexpressed in *Escherichia coli (BL21DE3)* strain, isolated, and then reconstituted with freshly synthetized pCA (coumaric acid) anhydride in 4 M urea buffer (Mihara et al., 1997; Kamikubo et al., 2007). The holoprotein was purified by column chromatography (DEAE Sepharose CL6B) and concentrated/washed by 10 kDa centrifugal filters several times.

An important requirement for photonic applications is the optical quality of the films. Layering and subsequent drying of protein suspensions normally leads to highly cracked samples, inappropriate for technical applications. To prevent cracking, some ballast materials, such as gelatin or PVA are used at relatively high concentrations (Vsevolodov, 2012), and the relative humidity is often fixed at the desired values by sandwiching and sealing the sample between glass plates (Hampp and Juchem, 2001). In our experiments, we chose to add low amounts of glycerol to the sample before drying. These treatments completely prevented crack formation, at the same time, kept the sample sufficiently humid without sandwiching, even at low relative humidity values, such as 33% humidity, the standard laboratory environment. According to our estimate based on optical multichannel analyzer experiments on bR (data not shown), the humidity inside the sample corresponded to a ca. 80–85% relative humidity value. During the Z-scan measurements and the absorption kinetic experiments, we controlled the environment to avoid any errors arising from the difference in humidity.

Each of the protein suspensions was first mixed with an 87% glycerol solution. For bR samples, glycerol constituted 10% of the mixture, while for PYP the ratio was lowered to 2% to achieve optimal homogeneity and viscosity of the film. The mixtures were first sonicated for 1–2 min to remove any microbubbles, then pipetted to a 200-μm thick microscope cover slide to form an approximately 5 mm diameter patch. The films were then left to dry under an extractor fume hood for at least 12 h. Before measurements, the protein films were sandwiched using an additional microscope cover slip. The cover slips were made of BK7 glass. For the pulsed-laser measurements, a sample thickness of 200 μm was achieved by embedding a 200-μm thick spacer between the glass slabs. Similarly, for the 405-nm CW measurement on PYP, a sample thickness of 200 μm was used. For the 543-nm CW experiment on bR, a thinner sample was made to accommodate the lowered average power of the applied laser. The environment’s relative humidity was 33% during each experiment, while the sample temperature was kept at 23°C.

For the OWLS measurements, the protein-glycerol mixtures were prepared using the same methods as for the Z-scan measurements. The mixtures were pipetted to an optical slab waveguide, and left to dry for at least 12 h before measurements.

For the single-wavelength absorption kinetics experiments, the protein-glycerol mixtures, pipetted onto glass slabs, were left to dry for at least 12 h, and the slabs were then placed into cuvettes in which we set the relative humidity at 33% with a saturated solution of magnesium chloride.

### OWLS Measurements

Throughout the experiments, grating-coupled (coupler grating width 1 mm, line density 2400 mm^–1^) slab waveguides [MicroVacuum Ltd., material Si(Ti)O_2_, ***n***_**f**_ between 1.78 and 1.80, thickness between 195 nm and 205 nm] on glass substrate (***n***_***s***_ = 1.53) were used. The waveguides were mounted on a high-precision rotational turntable (DPS, Ealing Electro Optics), by which the angle of incidence of a measuring light beam (He-Ne laser, Melles Griot, 543 nm) could be adjusted with an accuracy of 10^–4^ degrees. The intensity of the coupled light was detected at the end of the waveguide by a photomultiplier tube (PMT, Hamamatsu, Japan), whose signal was amplified by a laboratory-built current-voltage converter and recorded by a digital oscilloscope (LeCroy 9310-L). Under such conditions, only two discrete modes (transversal electric, “TE” and transversal magnetic, “TM”) of the guided light can propagate, with highly selective resonance conditions. From the peak positions, the refractive index of the adlayer can be determined using the mode equations of the three-layer waveguide (Ramsden, 1993).

### Absorption Kinetics Measurements

Time-resolved absorption spectra of the protein samples were obtained with a home-built pump-probe apparatus on the time scale of milliseconds to seconds. The pump source was a Surelite II Nd:YAG laser with an OPO extension (Continuum, United States) and was aligned to 514 nm and 445 nm for exciting the initial state of bR and PYP, respectively. The pulse energies were 2 mJ and 2.80 mJ. The source of the unpolarized probe light was a 35-W high-pressure Xenon lamp (Hamamatsu, Japan), filtered by narrow-band interference filters at selected wavelengths. The pump and probe beams spatially overlapped on the sample, and the transmitted probe light was directed to a Hamamatsu PMT through a HR-320 monochromator (ISA Jobin–Yvon, France). The applied repetition rate of the excitation laser was chosen to be 0.1 Hz to ensure that both proteins return to the initial state during each measuring cycle. The output signals of the photomultiplier were amplified by a home-made current-voltage converter, and recorded by a National Instruments oscilloscope card. In total, 10 traces were averaged.

Light-excitation experiments were also done to measure the difference spectrum of the protein state formed after illuminating the samples with a CW laser beam for a longer period. The experimental setup was the same as in the absorption kinetics measurements, except that the measuring light was filtered by a gray-colored glass filter before reaching the sample. The measuring light was detected with a spectrograph equipped with an iStar gated ICCD detector (Andor Technology, United Kingdom). For the light-excitation of the bR film, a Nd:YAG laser (DHOM-M-532-500 mW) was used with an average power of 5 mW at 532 nm wavelength, while for PYP, 40 mW at 410 nm was applied. The samples were illuminated for 10 s before the start of the measurements. 10 spectra were averaged, and each measurement lasted for 1.30 s to complete.

### Z-Scan Measurements

**FIGURE 1:**
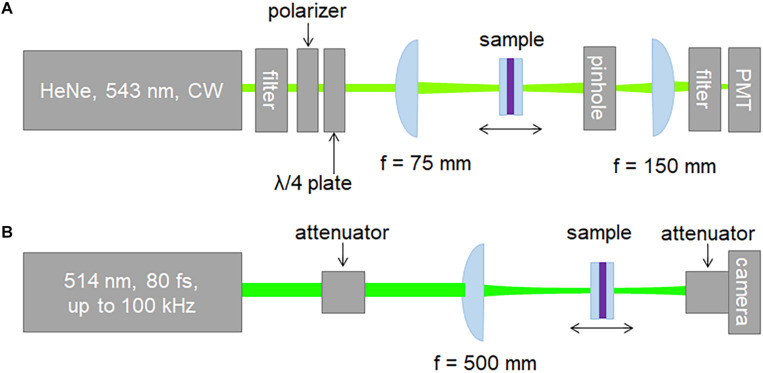
Schematics of the experimental **(A)** CW and **(B)** pulsed-mode Z-scan setup.

We utilized the Z-scan technique to characterize the nonlinear refractive index of dry bR and PYP films. Both CW and pulsed laser beams were employed to interrogate the samples. The setup used with CW illumination is shown in Figure 1A. The closed-aperture (CA) Z-scan traces were recorded by a PMT placed behind a circular aperture in the far-field, while the open-aperture (OA) traces were measured without an aperture in the laser beam. The light source was a 543-nm He-Ne laser delivering an average power of 0.75 mW. The power of the He-Ne laser beam was adjusted using a half-wave plate and a polarizer. A quarter-wave plate was placed between the polarizer and the sample to suppress back-reflection toward the laser. Accordingly, the laser beam incident on the samples was circularly polarized in the CW experiments.

The setup used with pulsed excitation was a modified version of the scheme described in detail elsewhere (Mero et al., 2019) and is shown in Figure 1B. Briefly, the laser source was a commercial Yb:KGW laser oscillator-amplifier system delivering 1.028-μm pulses at adjustable repetition rates up to 100 kHz. The fundamental pulses were frequency-doubled yielding linearly polarized 82-fs, 514-nm pulses for probing the samples. The temporal intensity profile of the second-harmonic pulses was characterized using the self-diffraction frequency-resolved optical gating (SD-FROG) technique. To reduce the peak power, the pulses were attenuated by reflecting them off an uncoated wedge and sending them through neutral density filters. Further adjustment of the peak power was achieved using a half-wave plate and a thin film polarizer. An *f* = 500-mm singlet lens was used to focus the pulses on the sample leading to a Gaussian beam waist radius of 37 μm measured with a beam profiling camera. The minor astigmatism of the laser beam was eliminated by an appropriate tilt of the focusing lens. The *M*^2^ values in the horizontal and vertical planes were measured to be ≤1.1. The pulsed Z-scan setup was also used to conduct CW Z-scan measurements at 405 nm, using a single longitudinal mode, TEM_00_-spatial-mode, temperature-stabilized diode laser (not shown in Figure 1B). In this case, the focusing conditions were changed leading to a Gaussian beam waist radius of 36.6 μm. In contrast to the standard implementation of the Z-scan measurement scheme relying on single-pixel detectors, we employed a beam profiling camera. The open- and closed-aperture Z-scan traces were extracted from the measured beam profiles using image processing. Namely, the OA traces were determined by adding the signal counts of all pixels of the camera. The CA traces can be obtained by adding the signal counts in a predefined 2D pixel area centered on the center of gravity of the beam profile, which mimics a real hard aperture in the laser beam.

For samples with absorptive nonlinearities, the CA traces become distorted compared to those containing only refractive nonlinearities. Elimination of such distortive effects on the CA traces is often possible by dividing the CA traces by the OA traces. This procedure in turn allows the use of simple fit functions applicable to purely refractive cubic nonlinearities and a straightforward extraction of the nonlinear refractive index of the sample even in the presence of nonlinear absorption. However, we found that this procedure is not applicable for our protein samples exhibiting strong nonlinear absorption. Relying on 2D camera-based detection in our Z-scan measurements, we developed a novel procedure to remove the distortions in CA traces even in the presence of massive nonlinear absorption (see section “Procedure for Separating Refractive Nonlinearities in Closed-Aperture Z-Scan Traces”). This procedure is also useful to significantly reduce the noise in CA traces for samples exhibiting spatial inhomogeneity.

After the removal of nonlinear absorption effects, we fitted the CA traces with the expression valid for a cubic nonlinearity, negligible nonlinear absorption, thin-samples, and a peak, on-axis phase shifts below π, according to Sheik-Bahae et al. (1990):

(1) $$T\left(x,\left\langle \Delta \phi_{0}\right\rangle \right)=1-\left[4x\left\langle \Delta \phi_{0}\right\rangle \right]/\left[\left(x^{2}+9\right)\left(x^{2}+1\right)\right].$$

**FIGURE 2:**
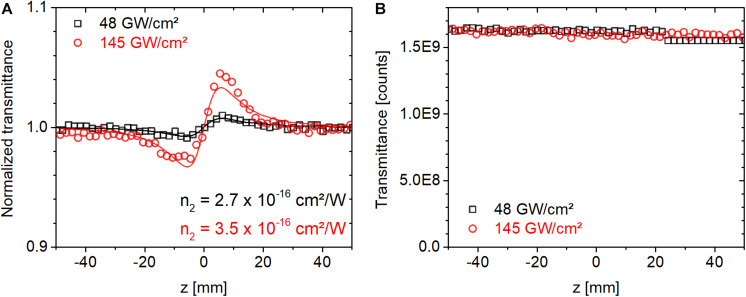
**(A)** Closed and **(B)** open-aperture Z-scan traces measured for a BK7 substrate using 514-nm ultrafast laser pulses at a repetition rate of 1 kHz at peak on-axis intensities of 48 and 145 GW/cm^2^.

In Eq. 1, $x=\frac{z}{z_{0}}$ is the sample position ***z*** normalized by the Rayleigh range ***z***_**0**_, while ⟨**Δϕ_0_**⟩ is the time-averaged, peak, on-axis phase shift. The ***n***_**2**_ values were calculated according to the formula,

(2) $$n_{2}≅\left\langle \Delta \phi_{0}\right\rangle \left(F\lambda w_{0}^{2}\right)/\left(L_{\mathrm{eff}}P\right),$$

where **λ** is the center wavelength, ***w*_0_** is the Gaussian beam waist, **L_*eff*_** = (1−**e^−α*L*^**)/**α** is the effective sample thickness, **α** is the linear absorption coefficient and ***L*** is the sample thickness. ***P*** is the laser power inside the material. For temporally Gaussian pulses ***P*** = ***E_P_***/**1.064⋅τ_*P*_**, or ***P** = **E**/**T_rep_***, when peak or average power is considered, respectively. Here **τ_*P*_** is the pulse duration at full-width of half-maximum, and ***T_rep_*** is the laser repetition period. The factor ***F*** is $\sqrt{2}/4$ or 1/4 when the peak or average power aspect of the illumination is considered to be relevant, respectively. In Eq. 2, ⟨**Δϕ_0_**⟩ is the time-averaged peak on-axis phase shift obtained from fitting the expression in Eq. 1 to experimentally measured CA traces. We note that we tested the accuracy of the pulsed Z-scan setup by measuring the ***n***_**2**_ value of a BK7 sample (an empty substrate without a protein film) and we obtained a value of ∼3 × 10^–16^ cm^2^/W at 514 nm in agreement with the literature (Adair et al., 1987; Nibbering et al., 1995; Boyd, 2003). Figure 2 shows the corresponding closed and open-aperture traces. We note that the fluctuations in Figure 2B, characterized by a standard deviation of 0.6%, are indicative of camera noise, as BK7 exhibits negligible nonlinear absorption at our applied peak intensities. We found that camera noise had a larger impact on the open-aperture traces than the closed-aperture traces as a result of a drift of the baseline values, which strongly affects the total signal count from the entire CMOS chip.

## Results and Discussion

### OWLS Measurements

High-resolution scans by the angle of incidence were carried out in the range of effective coupling, with the bare waveguide and, subsequently, waveguides coated with the biofilms. The recorded traces were fitted by Gaussians, in order to obtain the positions of the TE and TM modes. First, the actual refractive index and thickness values of the guiding layer were determined, then, using these data, the refractive index of the biofilms was obtained, making sure that the thickness of the adlayer was at least an order of magnitude larger than the penetration depth of the guided light (Ramsden, 1993). The measured and calculated values are summarized in Table 1. These refractive index data of the biofilms were used for the determination of their ****Δ***n*** and ***n***_**2**_ values from the Z-scan experiments. Adding glycerol to the protein solutions before drying them modifies the refractive indices of the thin film samples, which must be taken into account during the evaluation of the Z-scan data.

**TABLE 1:** The measured TM and TE positions and the calculated refractive index values of bR and PYP samples.

	TM position (degree)	TE position (degree)	Refractive index	Thickness
Bare waveguide 1	15.989	19.111	*n*_*f*_ = 1.788	205.45 nm
bR film	18.392	20.270	*n*_*bR*_ = 1.427	>5 μm
Bare waveguide 2	15.777	19.164	*n*_*f*_ = 1.799	195.26 nm
PYP film	18.672	20.595	*n*_*PYP*_ = 1.460	>5 μm

### Absorption Kinetics Spectroscopy

The absorption kinetics data of both bR and PYP were measured at 33% ambient relative humidity. The photocycles of both bR and PYP are sensitive to environmental parameters, such as temperature or pH, and they are vastly different in the proteins’ dry state at different relative humidity values. Therefore, we conducted all our measurements under the same relative humidity, for which we arbitrarily chose a value of 33% as stated in Materials and Methods. In the current study, glycerol was added to the protein suspensions before drying, which further modifies the photocycle, via changing the microviscosity of the medium (Beece et al., 1981), controlling water activity inside the films and enhancing hydrophobic interactions at the protein surface (Draheim and Cassim, 1985). To accurately determine the intermediates which the measured ***n***_**2**_ values belong to, and to see how the distinct intermediates form and decay in time, single-wavelength kinetics measurements were carried out on the proteins. The glycerol-doped films were of superior optical quality, showing reduced light scattering even for the membrane-bound bR, due to quasi-matching of its refractive index with that of bR-containing purple membranes (Ormos et al., 2002).

**FIGURE 3:**
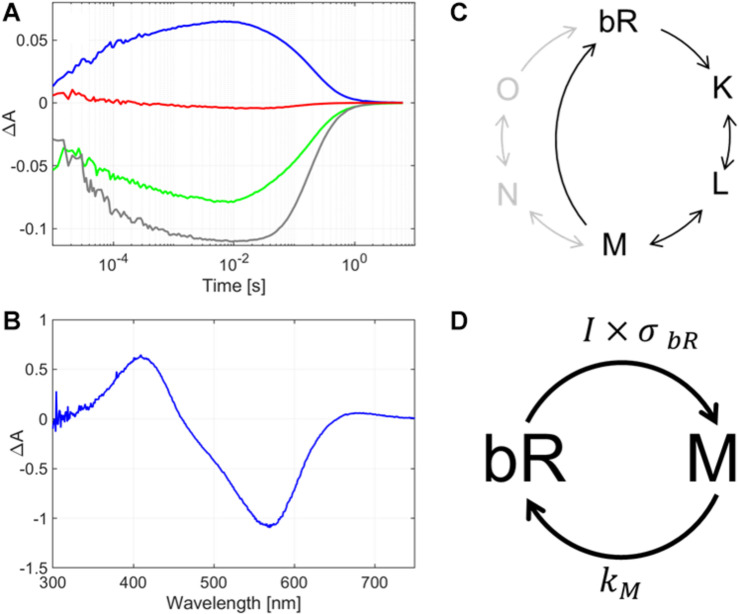
**(A)** Absorption kinetics and **(B)** intermediate accumulation measurements taken on a glycerol-containing bR film. In panel **(A)**, the lines represent the change of absorption after light-excitation measured at 410 nm (blue line), 500 nm (green line), 575 nm (gray line) and at 620 nm (red line). In panel **(B)**, the difference absorption spectrum is shown, as measured after a 10-s excitation by subsequent laser pulses of a repetition rate of 2 Hz. **(C)** Schematic diagram of the full photocycle of a glycerol-containing bR film (black) and bR in water (gray). **(D)** Simplified diagram of the photocycle of the glycerol-containing bR film showing only the states relevant for our pulsed Z-scan measurements. *I* is the (average) light intensity exciting the sample, σ*_*bR*_* is the absorption cross section of the bR molecule in the initial state, at the wavelength of excitation, and *k*_*M*_ is the rate constant of the M-decay.

For bR, four wavelengths were chosen to monitor the absorption changes in time, to get a comprehensive picture of the protein’s photocycle. These wavelengths were 575 nm to observe the changes corresponding to the initial state’s transient bleaching, 410 nm that corresponds to the absorption maximum of the M intermediate; and additionally 500 nm and 620 nm to gain spectral information for both the red- and blue-shifted side of the initial state’s maximum. We found that the dry bR-glycerol sample’s photocycle had a rate-limiting step of ca. 1 s relaxation time, which is considerably shorter than the rate-limiting steps in the dry bR film’s photocycle (Tóth-Boconádi et al., 2011) (Figure 3A). The most probable reason for this phenomenon is that the glycerol-mediated wetting effect on the bR-conforming purple membranes dominates over viscosity effects, which were supposed to decelerate the decay of M intermediate (Beece et al., 1981). By also measuring the absorption difference spectrum after 10 s of CW excitation, we found that the majority of the bR molecules accumulate in the M intermediate state (Figure 3B). Compared to the flash duration and the interflash intervals of the Z-scan measurements, the decay of the photocycle is orders of magnitude slower, even at the lowest repetition rate. At 10 ms after excitation – which corresponds to the interflash interval at the lowest repetition rate in our Z-scan measurements –, the bR molecules are accumulated in the M intermediate state. Since the absorption cross section of bR in the M intermediate state is negligible at the wavelength of excitation (514 nm), and in dry samples the other long-living intermediates of the normal bR-photocycle (N and O) do not accumulate in considerable amounts (Tóth-Boconádi et al., 2011), the subsequent flashes hitting the sample will initiate photoreactions of the remaining ground-state molecules only. Hence, the exciting laser-flash train in the pulsed Z-scan experiments can be considered a quasi-CW illumination, while the sample can be considered to be in an average-intensity dependent dynamic equilibrium mixture of bR molecules in M intermediate and the initial state. Figure 3C shows the schematic representation of the photocycle of bR, where the most relevant part (marked with black color) corresponds to the glycerol-containing bR film. For the higher repetition rate measurements, hence the exciting laser-flash train in the pulsed Z-scan experiments can be considered quasi-CW illumination, while the sample can be considered to be in an average-intensity dependent dynamic equilibrium mixture of bR molecules in the M intermediate and the initial state. The corresponding simplified photocycle relevant for our Z-scan measurements, too, is illustrated in Figure 3D, indicating only the rate-limiting reactions. Here, ***I*** is the (average) light intensity exciting the sample, and **σ*_***bR***_*** is the absorption cross section of the bR molecule in the initial state, at the wavelength of excitation. The product of the two, i.e., the probability of excitation, ***I⋅*σ*_***bR***_***, represents a virtual rate constant driving out the bR population from the initial state. Since the other transitions of the photocycle are much faster, practically only the M intermediate accumulates under (quasi-) steady state conditions, at an equilibrium concentration of [***bR***]_**0**_***I*σ_*bR*_**/*(****k***_**m**_ + ***I*⋅σ_*bR*_*)***, where ***[bR_**0**_]*** is the concentration of all the bR molecules (including the ones in the M and in the initial states), and ***k***_**M**_ is the rate constant of the M-decay. The difference spectrum of such a sample is shown in Figure 3B, and the calculated ****Δ***n*** values are related to this steady state.

**FIGURE 4:**
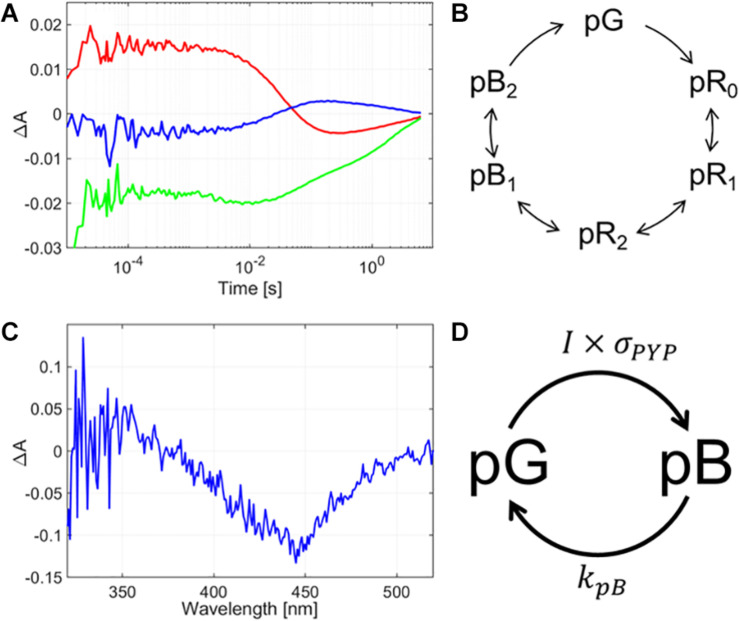
**(A)** Absorption kinetics measurements and **(B)** intermediate accumulation measurements taken on a glycerol-containing PYP film. In subfigure **(A)**, the blue line represents the absorption spectrum measured at 360 nm, the green line at 430 nm, and the red line at 470 nm. In panel **(B)**, the difference absorption spectrum is shown, as measured after a 10-s excitation of the sample by a CW laser of 410 nm and 40 mW. **(C)** Schematic diagram of the full photocycle of a glycerol-containing PYP film. **(D)** Simplified diagram of the photocycle of the glycerol-containing PYP film showing only the states relevant for our pulsed Z-scan measurements. *I* is the (average) light intensity exciting the sample, *σ_PYP_* is the absorption cross section of the PYP molecule in the initial state, at the wavelength of excitation, and *k*_PB_ is the rate constant of the pB-decay.

Similarly to bR, the wavelengths chosen for measuring the PYP’s absorption kinetics represent the concentration development of different intermediates of the photocycle. The photocycle was monitored at three selected wavelengths: 430 nm, which is slightly blue-shifted from the initial state’s maximum in order to limit the backscattering effect from the exciting light, while still gaining information about the initial state’s concentration changes; 360 nm, to monitor the pB intermediate, and 470 nm for the pR intermediates. The rate-limiting step of the PYP-glycerol film’s photocycle was found to be ca. 6 s (Figure 4A). Note that in PYP films devoid of glycerol, the photocycle does not take place at such low humidity, yet in our current measurements there is a clear indication of spectral changes in the wavelength range of the pR and pB intermediates, indicating a photocycle still being present in the glycerol-doped films (Figure 4C). Based on the glycerol concentration, the water activity in the sample was estimated to be the same as at 80% relative humidity without glycerol (Wheeler et al., 2012). Nevertheless, the photocycle is significantly slower than without glycerol (Krekic et al., 2019), which may be attributed to viscosity effects (Beece et al., 1981) rather than water-structure-mediated kosmotropic effects (Násztor et al., 2016), which should destabilize open conformations, such as pB, hence they should accelerate its decay (Kamikubo et al., 2007; Khoroshyy et al., 2013). Note that PYP is a water-soluble protein, unlike the membrane-protein, bR, so it is more exposed to solvent viscosity effects. The accumulation measurements (Figure 4B) are indicating a majority of the protein present in the pB state after excitation, hence, similarly to bR, we can suppose PYP is driven to a steady-state of pB and initial states, as indicated in Figure 4D, where their ratio is adjusted by the average exciting light intensity applied during pulsed Z-scan measurements (see similar argumentation above, for bR).

### Z-Scan Measurements

#### Procedure for Separating Refractive Nonlinearities in Closed-Aperture Z-Scan Traces

**FIGURE 5:**
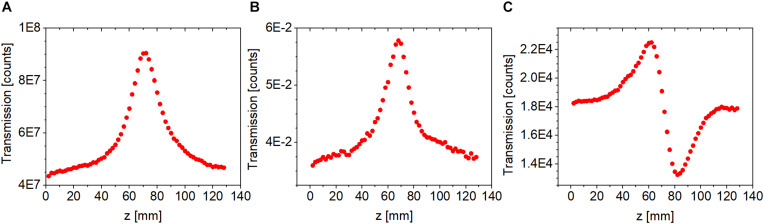
Closed-aperture Z-scan traces measured using fs laser pulses at 514 nm for a bR sample at an average peak intensity of 0.13 W/cm^2^. **(A)** Uncorrected closed-aperture trace. **(B)** Corrected closed-aperture trace obtained by dividing the uncorrected closed-aperture trace (cf. **A**) by the corresponding open-aperture trace. **(C)** Corrected closed-aperture trace obtained by applying our novel method described in Section “Procedure for Separating Refractive Nonlinearities in Closed-Aperture Z-Scan Traces.”

Saturable absorption can drastically alter CA Z-scan traces even if the laser intensity does not significantly exceed the saturation intensity (cf. Figure 5A). Under such circumstances, the standard approach of dividing the CA trace by the corresponding OA trace is not sufficient to eliminate the effect of nonlinear absorption on the CA trace (cf. Figure 5B). Enabled by the use of a 2D camera in our Z-scan measurements, such effects can be removed using the following procedure. First, a 2D Gaussian spatial distribution is fitted on the measured beam profiles at each ***z*** position, and the position dependent beam waists, ***w_x_***(***z***) and ***w_y_***(***z***), and amplitudes, ***A(z)***, are extracted. Then, the amplitudes are corrected assuming that the background-subtracted total signal count (E, energy) remains constant, i.e., independent of ***z***. This constant is defined at a ***z*** position where nonlinear absorption is negligible,

(3) $$E=A\left(|z|\gg z_{0}\right)\frac{w_{x}\left(|z|\gg z_{0}\right)w_{y}\left(|z|\gg z_{0}\right)\pi }{2}=constant.$$

The corrected amplitudes, **A**′(**z**), constitute the corrected CA trace with an infinitesimally small aperture (cf. Figure 5C). In addition to separating nonlinear absorption effects from the CA traces, this correction procedure also reduces the fluctuations due to, e.g., sample inhomogeneity. All CA traces shown below are corrected traces based on this procedure.

#### Nonlinear Refractive Index of bR

**FIGURE 6:**
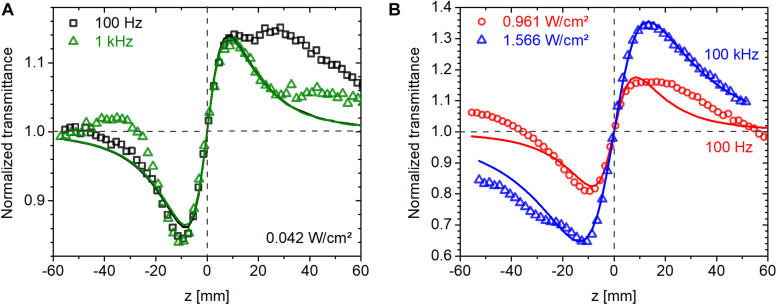
Closed-aperture Z-scan traces measured on bR using fs laser pulses at 514 nm at average intensities of **(A)** 0.042 W/cm^2^ and **(B)** around 1 W/cm^2^. The symbols are measured data points obtained at various laser repetition rates, and the lines are fitted traces based on Eq. 1.

Three laser repetition rates were used at 514 nm, 100 Hz, 1 kHz, and 100 kHz, to investigate the role of thermal effects and possible variation in the population distribution among various states in the photocycle of bR. CW measurements were also conducted at comparable intensities at 543 nm. Since the photocycle of bR is much longer than the laser repetition period (see section “Absorption Kinetics Spectroscopy”), the material is expected to be in a steady state, where a significant fraction of the molecules is in the M intermediate state (cf. Figure 3D). Also, the nonlinear response is expected to be driven by the average intensity rather than the peak intensity. In agreement, as shown in Figure 6A, we found that the magnitude of nonlinear refraction at an average intensity of 0.042 W/cm^2^ was the same at a repetition rate of 100 Hz and 1 kHz despite an order of magnitude difference in peak intensity. At higher intensities, we found this to be still approximately true. The CA traces shown in Figure 6B were recorded at average intensities of 0.96 and 1.57 W/cm^2^ at 100 Hz and 100 kHz, respectively. The ratio of **Δ***T*_P–V_ (the difference between normalized peak and valley transmittance) of the 100-Hz and 100-kHz traces is 0.52, while the average intensity ratio is 0.61, i.e., only 18% higher than expected. In contrast, the ratio of peak intensities is 610.

**FIGURE 7:**
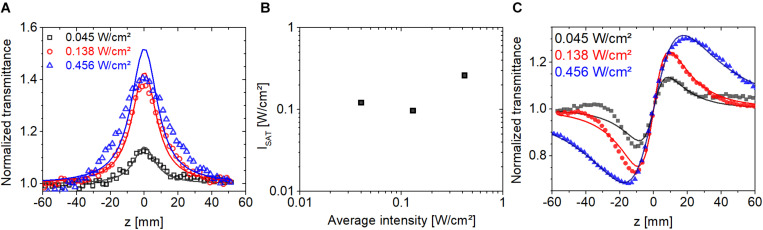
**(A)** Open-aperture Z-scan traces measured on bR using fs laser pulses at 514 nm at three excitation intensities at a repetition rate of 1 kHz. **(B)** Saturation intensities extracted from the open-aperture traces in panel **(A)**. **(C)** Closed-aperture Z-scan traces obtained at 514 nm excitation corresponding to the curves in panel **(A)**. The symbols are the measured data points, and lines show the fitted traces.

To explore the saturable absorption behavior, we recorded open and closed-aperture Z-scan traces as a function of average intensity in the range of 0.04 and 0.42 W/cm^2^ at a laser repetition rate of 1 kHz. The sample was 200 μm thick with an OD of 0.35 at 514 nm. Based on (Gu et al., 2006), we modeled the OA traces by assuming saturable absorption of a homogeneously broadened resonant transition characterized by an absorption coefficient,

(4) $$\alpha \left(I\right)=\frac{\alpha_{0}}{1+\frac{I}{I_{s}}},$$

where α*_**0**_* is the linear absorption coefficient, and ***I***_***s***_ is the saturation intensity. The results of the modeling are shown in Figure 7A. At the two lowest intensities, the fit is relatively good but it gets gradually worse at higher intensities. The extracted saturation intensities are shown in Figure 7B with a value approximately 0.1 W/cm^2^ at an excitation intensity of 0.04 W/cm^2^. The estimated error for the value of ***I***_**s**_ at the lowest average intensity is ±20%, which includes uncertainty in the measured value of sample absorbance, thickness, and camera signal. The corresponding CA traces are shown in Figure 7C. At the two lowest intensities, the peak-to-valley distances are approximately the same with a value of ∼***2z***_**0**_, while at the highest intensity it is significantly larger. According to the Z-scan theory with cubic refractive nonlinearity (Sheik-Bahae et al., 1990), the peak-to-valley distance is ∼***1.7z***_**0**_ and it remains nearly constant at higher intensities with a small gradual decrease with increasing intensity. Therefore, at excitation intensities below 0.1 W/cm^2^, we consider Eq. 1 a satisfactory model of nonlinear refraction of our bR samples.

**FIGURE 8:**
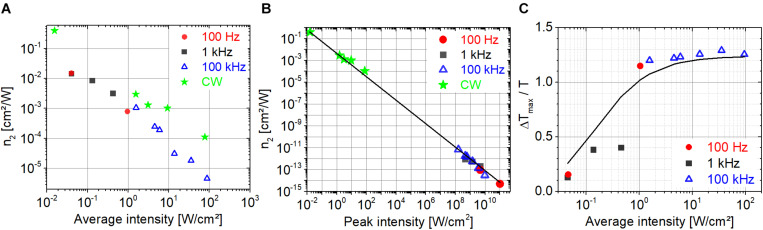
Nonlinear refractive index of bR at 514 nm as a function of **(A)** average and **(B)** peak intensity. **(C)** The relative change in transmission as a function of average intensity. The solid line is a fit of a saturable absorption model on all measured data.

In Figures 8A,B, the nonlinear refractive index, ***n***_**2**_, is shown in a broad range of intensities. Although, we do not expect extracted ***n***_**2**_ values to be consistent with the approximations implied by Eq. 1, our goal was to compare our measured ***n***_**2**_ values with those in the literature and to provide for us benchmark values for the investigation of PYP (see section “Nonlinear Refractive Index of PYP”). Our measured ***n***_**2**_ values in the average intensity range of 10^–2^ to 10^2^ W/cm^2^ fall in the range of 10^–1^ to 10^–5^ cm^2^/W (Figure 8A) in agreement with the literature on bR excited in the green spectral range by CW lasers (Song et al., 1993; Kir’Yanov et al., 2000; Sifuentes et al., 2002; Banyal and Raghavendra Prasad, 2007). The increasing gap with increasing average intensity between the ***n***_**2**_ values measured with a CW and a pulsed laser may suggest that further processes, other than a simple cubic nonlinearity are at play, such as thermal effects, thermally induced conformational changes, and refractive or absorptive nonlinearities of higher order. Our ***n***_**2**_ data plotted as a function of peak intensity are also consistent with the literature on both bR and different retinal derivatives excited by pulsed lasers (Bezerra et al., 1997; Rakovich et al., 2013). In our peak intensity range of 10^8^ to 10^11^ W/cm^2^, the ***n***_**2**_ values are in the range of 10^–11^ − 10^–14^ cm^2^/W. These ***n***_**2**_ values are many orders of magnitude smaller than the values obtained using CW lasers, which is typically not noted or discussed in the literature. As the average intensity is the main driving force behind the refractive nonlinearities of bR even at relatively low repetition rates (down to 100 Hz in our studies), we think that it is more relevant to determine and quote ***n***_**2**_ values based on average intensities even for pulsed lasers. Interestingly, our ***n***_**2**_ data closely follow an intensity dependence characterized by ***I***^–1.075^, where ***I*** is the peak intensity (cf. solid line in Figure 8B). Importantly, the ***n***_**2**_ data measured by our CW laser are also on this trend line. Accordingly, the corresponding change in refractive index, **Δ*n* = *n_**2**_I***, in the whole intensity range is approximately constant with a value of 0.0009 to 0.0011 at 514 nm and around 0.0066 at 543 nm. This **Δ*n*** value is in agreement with the refractive index change corresponding to the transition from the bR initial state to the M state (Song et al., 1993; Ormos et al., 2002; Fábián et al., 2015).

The saturable absorption behavior is also driven by average intensity rather than peak intensity. Figure 8C shows the relative change in the normalized transmission at ***z* = 0** as a function of average intensity approximately following the saturation behavior of a homogeneously broadened transition (solid line). Obviously, the data do not follow a single trend when plotted as a function of peak intensity (not shown).

#### Nonlinear Refractive Index of PYP

In contrast to bR, excitation of the initial state of PYP (pG) at 514 nm is expected to lead to negligible linear absorption (see Figure 4). Therefore, the photocycle is not expected to be triggered at low excitation intensities. If the photocycle is initiated at laser repetition rates ≥1 kHz, it would lead to the accumulation of the pR intermediate state. Once the pR intermediate is produced, all the subsequent intermediates and also pR will linearly absorb at 514 nm.

**FIGURE 9:**
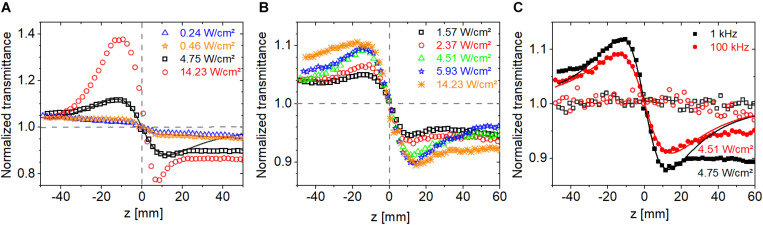
Closed-aperture Z-scan traces measured on PYP using fs laser pulses at 514 nm at different excitation intensities at a repetition rate of **(A)** 1 kHz and **(B)** 100 kHz. **(C)** Comparison of the closed-aperture traces (filled symbols) and the open-aperture traces (hollow symbols) at a repetition rate of 1 kHz and 100 kHz, respectively, at ∼4.6-W/cm^2^ excitation intensity.

We recorded Z-scan traces using pulsed-excitation at a repetition rate of 1 kHz and 100 kHz at 514 nm. Figures 9A,B show the CA traces measured at 1 kHz and 100 kHz, respectively, in the average intensity range of 10^–1^ and 10^1^ W/cm^2^. Similarly to bR, the nonlinear response of PYP is also driven by the average intensity rather that peak intensity. In contrast to bR, the peak-to-valley configuration in the CA traces indicates a defocusing nonlinearity (i.e., negative ***n***_**2**_). Note that the negative sign is in concert with the negative ****Δ***n*** measured at 633 nm in Krekic et al. (2019) and by Lee et al. (2018) between 470 nm and 570 nm. Figure 9C shows the CA traces measured at an average intensity of ∼4.6 W/cm^2^ at 1 kHz and 100 kHz. Despite of a factor of 100 larger peak intensity at 1 kHz than at 100 kHz, the ****Δ*****T*_P–V_ values are within ∼30% suggesting that the excitation mechanism is not two-photon absorption but possibly a weak linear absorption process. In agreement, the OA traces show no sign of nonlinear absorption (cf. Figure 9C open symbols). With linear absorption, the photocycle can be started, and we expect the related refractive index changes to be larger than possible thermo-optical contributions.

**FIGURE 10:**
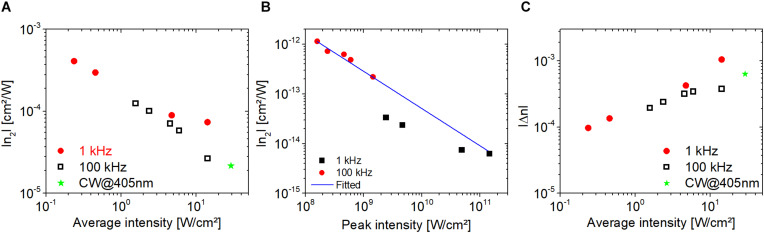
Absolute value of the nonlinear refractive index of PYP at 514 nm as a function of **(A)** average and **(B)** peak intensity. **(C)** The absolute value of the change in the refractive index as a function of average intensity.

The nonlinear refractive index of PYP extracted from the CA traces in Figure 9 is shown in Figures 10A,B. The absolute value of our measured ***n***_**2**_ data is also inversely proportional to the average intensity as in the case of bR (cf. Figure 8A), and these values fall in the range of 10^–3^ to 10^–4^ cm^2^/W (Figure 10A) in the average intensity range of 0.1 to 1 W/cm^2^. To the best of our knowledge, these are the first ***n***_**2**_ values provided for PYP. If one extracts the ***n***_**2**_ values from the ****Δ***n*** data provided in Vanhanen et al. (1998), the range of −2 × 10^–2^ to −9 × 10^–3^ cm^2^/W is obtained for an average intensity between 3 × 10^–3^ and 2 × 10^–2^ W/cm^2^ at 475-nm CW laser excitation. When plotted as a function of peak intensity, the ***n***_**2**_ data points follow different power laws at different laser repetition rates (Figure 10B). The solid line was obtained by fitting the nonlinear refractive index data points at 100 kHz. Clearly, the 1 kHz data points do not follow this trend. At both repetition rates, the dependence as a function of peak intensity is weaker than in the case of bR. The magnitude of the ***n***_**2**_ values of PYP in the peak intensity range of 10^8^–10^11^ W/cm^2^ are between 10^–12^ and 10^–14^ cm^2^/W, which is a factor of 10 smaller range than that obtained with bR. Accordingly, the magnitude of the ****Δ***n*** values at the two repetition rates is not constant, but also increases as a function of average intensity, following a weak power law (cf. Figure 10C). In general, the ****Δ***n*** values obtained at a particular repetition rate and average intensity were of the same order of magnitude for both bR and PYP. However, in contrast to PYP, the dependence of the ****Δ***n*** values of bR as a function of average intensity is not monotonous and can be considered constant centered at 9 × 10^–4^ in a full range of 5 × 10^–4^ to 2 × 10^–3^ (not shown). The range of ****Δ***n*** values we obtained for PYP is in good agreement with the range obtained with 475-nm CW excitation, where the photocycle can unambiguously be initiated (Vanhanen et al., 1998).

#### Nonlinear Refraction as a Result of the Photocycle vs. Thermo-Optic Effects

Dealing with protein-containing samples, one should always consider the possibility of thermo-optic effects, reported for light-harvesting proteins, such as LHC complexes (Garab et al., 2002) or bR, too (Dancsházy et al., 1999). Such effects were shown to have a regulatory role in photosynthesis (Garab et al., 2002). In bR, it was demonstrated that high-energy flashes in the main absorption band of the protein can lead to permanent photobleaching of the sample. The effects were interpreted as a consequence of the transient increase of the temperature in the vicinity of the absorbing chromophore by more than 10 degrees, giving rise to temperature-induced conformational changes in the proteins. The dissipation of the heat packet was estimated to take place in the nanosecond time regime, and the effects showed a strongly nonlinear dependence on the peak intensity of the flash, attributed to a threshold phenomenon (Dancsházy et al., 1999). In the present measurements, however, we do not see such an effect as a function of peak intensity (this is why both the ****Δ***n*** and the ***n***_**2**_ dependences measured at different peak intensities could be reasonably unified to common curves on the time-averaged intensity scale), neither experienced we any photobleaching of the samples. Note, that the pulse energies were also much lower in our measurements than in Dancsházy et al. (1999). Of course, an effect of global heating of the sample at high average exciting energies cannot be excluded here, either, supporting our assumption that the reliable values for these parameters are those in the low average power regime.

## Conclusion

Our glycerol-doped thin films proved to be appropriate for optical applications: they allow to achieve a high protein concentration and optical quality, at the same time control the necessary water activity for the proteins to undergo a photocycle. They also show proper stability. A comprehensive study of the NLO properties of the protein-containing films was performed, by characterizing their absorption kinetics properties from the 10 μs to the 10 s time scale, and determining their linear and nonlinear refractive indices (***n***_**0**_, ****Δ***n*,** and ***n***_**2**_ values).

The novel evaluation procedure we used for the Z-scan experiments could properly account for saturation effects that allows the determination of ***n***_**2**_ values for a Kerr material with much higher precision and reliability than before. At low average intensities, i.e., <0.1 W/cm^2^, our obtained **Δ*n*** values, ∼10^–4^ for PYP and ∼10^–3^ for bR, are comparable to those of the best solid-state materials. Importantly, our ***n***_**2**_ values, 4 × 10^–4^ cm^2^/W for PYP and 10^–2^ cm^2^/W for bR, are several orders of magnitude higher than those reported earlier for inorganic NLO materials (Adair et al., 1989; Kulagin et al., 2003; Eggleton et al., 2011), highlighting the potential utilization of these chromoproteins in special applications of photonics, where high ****Δ***n*** and ***n***_**2**_ are required. Some promising experiments have already been carried out in the fields of dynamic polarization holography (Hampp et al., 1990; Burykin et al., 2011), image filtering (Castillo et al., 2001), all-optical switching (Ormos et al., 2002; Topolancik and Vollmer, 2006; Fábián et al., 2010, 2011; Roy et al., 2010; Korposh et al., 2018) but a plethora of new applications can be envisaged in all-optical signal processing, utilizing the ultrafast transitions of the photocycle (Fábián et al., 2011), e.g., in multi-wave mixing, nonlinear mode-coupling in fibers (Xu et al., 2015), and wavelength multiplexing-demultiplexing in IO devices.

## Data Availability Statement

The raw data supporting the conclusions of this article will be made available by the authors, without undue reservation.

## Author Contributions

SK, ZH, MM, LZ, and AD created the research idea. ZH and MM designed the pulsed Z-scan measurements. SK, SV, LZ, and AD designed and conducted the CW Z-scan measurements, the OWLS, and kinetic experiments. TZ and ZG prepared the PYP protein while SK prepared all the thin protein film samples. The OWLS and kinetic data were analyzed by SK and AD, while the Z-scan measurements were analyzed and fitted by ZH and MM. SK, MM, ZH, and AD wrote the manuscript. All authors discussed and revised the article.

## Conflict of Interest

The authors declare that the research was conducted in the absence of any commercial or financial relationships that could be construed as a potential conflict of interest.
